# Postponed Dental Visits during the COVID-19 Pandemic and their Correlates. Evidence from the Nationally Representative COVID-19 Snapshot Monitoring in Germany (COSMO)

**DOI:** 10.3390/healthcare9010050

**Published:** 2021-01-05

**Authors:** André Hajek, Freia De Bock, Lena Huebl, Benedikt Kretzler, Hans-Helmut König

**Affiliations:** 1 Department of Health Economics and Health Services Research, Hamburg Center for Health Economics, University Medical Center Hamburg-Eppendorf, 20251 Hamburg, Germany; b.kretzler.ext@uke.de (B.K.); h.koenig@uke.de (H.-H.K.); 2 Federal Centre of Health Education, 50825 Cologne, Germany; freia.debock@bzga.de; 3 Department of Tropical Medicine, Bernhard Nocht Institute for Tropical Medicine, University Medical Center Hamburg-Eppendorf, 20251 Hamburg, Germany; l.huebl@uke.de

**Keywords:** COVID-19, coronavirus, dental care, dental health services, dental visits, SARS-CoV-2, dental service use, postponed dental visits, check-up, dental examination, pain, dental complaints, oral health

## Abstract

(1) Background: The COVID-19 pandemic is accompanied by various societal and economic challenges. Furthermore, it is associated with major health challenges. Oral health is a key component of health. Therefore, both curative and preventive dental visits are important during pandemics. Since there is a lack of nationally representative studies focusing on postponed dental visits and their correlates during the COVID-19 pandemic, we aimed to fill this gap in knowledge; (2) Methods: Cross-sectional data (wave 17) were collected from a nationally representative online-survey (COVID-19 Snapshot Monitoring in Germany (COSMO)) conducted in July 2020. The analytical sample consisted of 974 individuals (average age was 45.9 years (SD: 16.5, from 18 to 74 years)). The outcome measure was postponed dental visits since March 2020 (yes; no) due to the COVID-19 pandemic. Furthermore, the type of postponed dental visits was recorded (check-up/regular dental examination; pain/dental complaints; planned therapy); (3) Results: 22% of participants reported to have postponed dental visits due to the COVID-19 pandemic since March 2020, whereas 78% of individuals did not report postponed visits (“no, attended as planned”: 29.2%; “no, examining pending”: 44.9%; “no, other reasons”: 3.9%). Among individuals who reported postponed dental visits, 72% postponed a “check-up/regular dental examination”, whereas 8.4% postponed a dental visit despite “pain/dental complaints” and 19.6% postponed “planned therapy”. Furthermore, multiple logistic regressions showed that the likelihood of postponed dental visits was positively associated with being younger (aged 65 and older, OR: 0.43, 95% CI: 0.22–0.85; compared to individuals 18 to 29 years), and higher affect regarding COVID-19 (OR: 1.36, 95% CI: 1.13–1.64); (4) Conclusions: Our study showed that more than one out of five individuals postponed a dental visit—particularly check-ups and regular dental examination—due to the COVID-19 pandemic since March 2020. Several correlates of these postponed visits have been identified. This may help identify and address individuals at risk for deterioration of oral health amplified by postponed dental visits.

## 1. Introduction

Access to regular dental visits is important to avoiding oral diseases [1,2]. Nevertheless, it should be noted that avoiding or postponing dental visits is frequent in Germany [3,4]. For example, this behavior could lead to periodontitis and caries lesions which could ultimately result in tooth loos [5]. Furthermore, postponed dental visits can additionally affect quality of life [6]. Consequently, poor oral health can decrease functional health [7].

Previous studies have focused on determinants of nonattendance and dental treatment avoidance [4,8], rather than postponement of dental visits as the outcome measure. Furthermore, studies determined postponement for financial reasons [3]. For example, it has been shown that dental anxiety is associated with avoidance behavior [8]. Moreover, it has been demonstrated that avoidance of dental treatment is associated with younger age, lower social status, unemployment, and decreased health (in terms of increased physical illnesses and increased depressive symptoms) [4].

Existing studies focused on nonattendance, avoidance, or postponement of dental visits prior to the COVID-19 pandemic. Thus far, one economic analysis using a modelling approach exists focusing on the impact of COVID-19 on dental practices [9]. A telephone-based survey conducted from 24 March to 2 April 2020 (146 German dentists) [9] showed that mitigation/suppression decreased use of dental services, particularly prevention (−80% in mean), periodontics (−76%), and prosthetics (−70%). According to Schwendicke et al., COVID-19 and associated policies had an economic impact on dental practices in Germany [9]. Comparably, a study conducted in China (Beijing) from 1 February to 10 February 2020 showed that the COVID-19 pandemic significantly decreased the use of emergency dental services (e.g., 38% fewer patients had emergency dental visits at the beginning of the COVID-19 pandemic compared to one month prior to the pandemic) [10]. During the same period, the proportion of oral and dental infections significantly increased [10].

However, up to now, nationally representative studies focusing on postponed dental visits (in general, rather than directly cost-related) and its correlates are lacking. We aimed to fill this gap in knowledge.

To put our findings into context, in Germany, corona measures such as school closings or closing of daycare centers were implemented on 16 March 2020. A week later (22 March 2020), public restrictions and travel bans followed. These measures were prolonged in subsequent weeks. Restrictions were loosened on the 20 April 2020. In the beginning of May, schools gradually reopened. In May, additional restrictions were loosened (e.g., playgrounds reopened and contact bans loosened). Further restrictions eased in June. Nevertheless, a spike in COVID-19 cases could lead to a reimplementation of regulations.

It is necessary to describe key characteristics of the German healthcare system. Health insurance is compulsory in Germany. Approximately 9 out of 10 individuals are members of the social statutory health insurance (SHI), solely 1 out of 10 individuals has private health insurance (PHI). Predominantly, civil servants, employed individuals exceeding a defined income threshold, and self-employed individuals can opt for PHI. Both categories of health insurance (PHI and SHI) cover most expenses of outpatient treatment (even for dental care services) in Germany. Access to health care is commonly guaranteed for all insured individuals. However, additional dental services (e.g., gold or ceramic inlays) which have an unproven medical benefit are usually not covered in SHI. It should be emphasized that waiting periods are relatively short in Germany [11,12]. Passon et al. give further insight into the German health care system [13]. With regard to the COVID-19 pandemic, it should be noted that routine dentistry was allowed to continue in Germany. It was therefore not restricted to emergency appointments.

## 2. Materials and Methods

### 2.1. Sample

Cross-sectional data were collected from wave 17 of the COVID-19 Snapshot Monitoring (COSMO) [14]. Solely in wave 17 individuals were asked about postponed dental visits.

The COSMO study started in early March 2020 (3rd/4th March) with weekly follow-up waves until 26 May. Afterwards, the survey continued in a 14-day interval. Wave 17 was conducted from 21st to 22nd of July 2020. In wave 17, *n* = 1001 individuals aged 18 to 74 years participated. Individuals younger than 18 years and individuals older than 74 years were excluded in this wave.

A market research company (Respondi) conducted the recruitment of participants from an online panel matching distribution of age, gender (crossed-quota: age x gender), and federal state (uncrossed) within the German population [15]. A large sample size was chosen to also detect small effects in the COSMO study [16].

Informed consent was obtained from all individual participants included in the study. Ethical approval for COSMO was obtained by University of Erfurt’s IRB (#202000302). All procedures performed in the COSMO studies involving human participants were in accordance with the ethical standards of the University of Erfurt institutional research committee and with the 1964 Helsinki Declaration and its later amendments or comparable ethical standards.

### 2.2. Dependent Variables

In concordance to other large cohort studies (e.g., Survey of Health, Ageing, and Retirement in Europe) individuals were first asked whether they had postponed a dental visit since March 2020 due to the COVID-19 pandemic (1 “Yes”, 2 “No, attended as planned”, 3 “No examination pending”, 4 “No, other reasons”). The outcome measure was dichotomized (0 = no, not postponed; 1= yes, postponed). Additionally, individuals were asked about the type of postponed dental visit (1 = “check-up/regular dental examination”, 2 = “pain/dental complaints”, and 3 = “planned therapy”).

A pretest with *n* = 14 individuals confirmed high face validity of our outcome measures.

### 2.3. Independent Variables

Various determinants were included in our study: sex, age group (distinguishing between: 18 to 29 years; 30 to 49 years; 50 to 64 years; 65 years and above), relationship/marriage (no; yes), presence of children under 18 years (no; yes), living arrangement (two or more individuals in the same household; living alone), migration background (no; yes), status of self-employment (no; yes), educational level (up to 9 years/10 years and more (without general qualification for university entrance); 10 years and more (with general qualification for university entrance)), region (East Germany; West Germany), town size (municipality/small town (1–20,000); medium sized town (20,001–100,000); small city (100,001–500,000); big city (>500,000)), COVID-19 cases/100,000 population (below median; above median), and chronic diseases (no; yes).

With regard to COVID-19, individuals were asked to rate how they were affected (consisting of seven items, seven-point scale). For instance, items were: “For me, the new type of corona virus is” ... “near” (1) to “far away” (7) or “inflated in media” (1) to “not given enough attention in media” (7) or “Something I keep thinking about” (1) to “Something I almost never think about” (7).

The total score was built by averaging items. In our study, Cronbach’s alpha was 0.78. Moreover, participants were asked to rate the severity of COVID-19 disease (“How do you assess an infection with the novel corona virus for yourself?”, from 1 = completely harmless to 7 = extremely dangerous).

### 2.4. Statistical Analysis

Sample characteristics (analytical sample) were first calculated stratified by postponement of dental visits (no; yes). Afterwards, multiple logistic regressions were performed to identify determinants of postponed dental visits due to the COVID-19 pandemic. In further analysis, we used a multinomial logistic regression (with “Yes, postponed dental visits” as the base outcome). Statistical significance was set at *p* < 0.05. *p* values between 0.05 and 0.10 were considered as marginally significant. Statistical analyses were performed using Stata 16.0 (Stata Corp., College Station, TX, USA).

## 3. Results

### 3.1. Sample Characteristics

Sample characteristics for our analytical sample (*n* = 974) are shown in Table 1. In the total sample, the average age equaled 45.9 years (SD: 16.5, from 18 to 74 years) with 51.1% of individuals being female. Postponed dental visits were associated with being female, age category, and affect regarding COVID-19. Further details are shown in Table 1.

**Figure 1:**
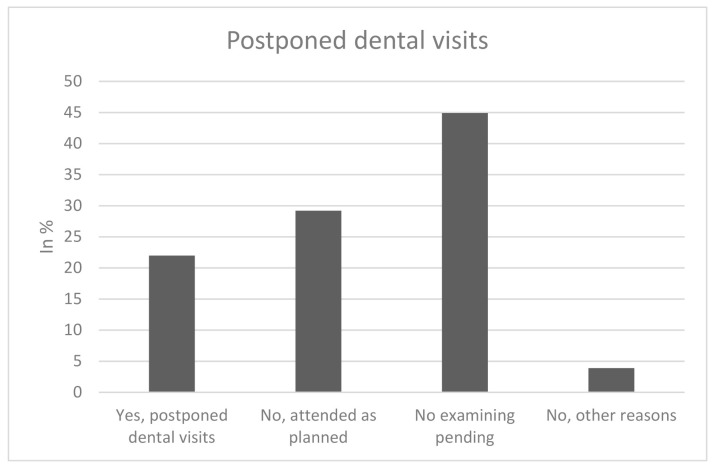
Postponed dental visits.

**Figure 2:**
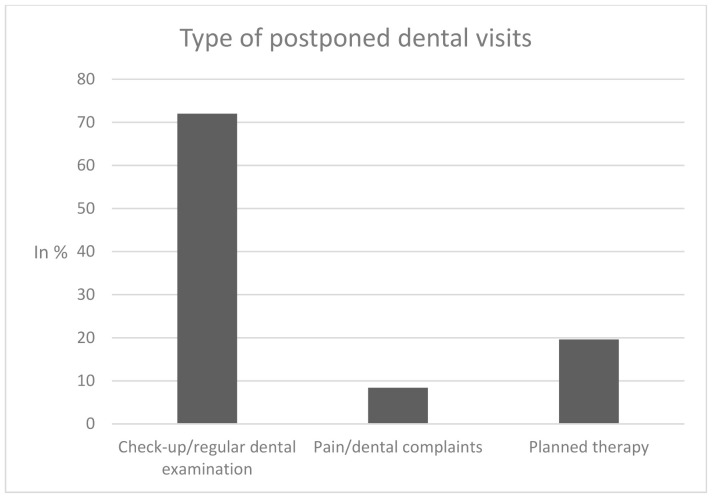
Type of postponed dental visits.

In sum, 22% of participants reported to have postponed dental visits due to the COVID-19 pandemic since March 2020, 78% did not report postponed visits (“no, attended as planned: 29.2%; “no, examining pending”: 44.9%; “no, other reasons”: 3.9%), as shown in Figure 1. Of the individuals who reported postponed dental visits, 72% postponed a “check-up/regular dental examination”, whereas 8.4% postponed a dental visit despite “pain/dental complaints” and 19.6% postponed “planned therapy” (Figure 2).

### 3.2. Regression Analysis

Multiple logistic regressions with postponed dental visits (0 = no, not postponed; 1 = yes, postponed) as outcome measures are displayed in Table 2. Regressions revealed that the likelihood of postponed dental visits due to the COVID-19 pandemic since March 2020 was positively associated with being younger (aged 65 and older, OR: 0.43, 95% CI: 0.22–0.85; compared to individuals 18 to 29 years), and higher affect regarding COVID-19 (OR: 1.36, 95% CI: 1.13–1.64). Furthermore, there was a marginal significant positive association between postponed dental visits and big cities (compared to small towns, OR: 1.53, 95% CI: 0.99–2.34). The remaining variables were not significantly associated with the outcome measure.

The results of further analysis with multinomial logistic regression (with “Yes, postponed dental visits” as the base outcome) are displayed in Supplementary Table S1. Findings remained comparable to our findings using multiple logistic regressions.

## 4. Discussion

Based on nationally representative cross-sectional data, the aim of this study was to clarify the frequency of postponed dental visits due to the COVID-19 pandemic and to determine its associated factors. Furthermore, the type of postponed dental visits was displayed (check-up/regular dental examination; pain/dental complaints; planned therapy). Based on individuals who postponed dental visits or did not attend as planned, it should be emphasized that approximately 43% of individuals postponed dental visits, and a significant amount postponed dental visits despite “pain/dental complaints”. Our study extends previous knowledge focusing on actual use of dental services in early 2020 [10] or modeled use of dental services [9].

Our study showed that more than one out of five individuals postponed a dental visit due to the COVID-19 pandemic between March and July 2020, particularly check-ups and regular dental examination. Predominantly individuals aged 30 to 49 years (29.1%) postponed dental visits. Regressions revealed that the likelihood of postponed dental visits was positively associated with being younger and higher affect regarding COVID-19.

Younger individuals are at an increased risk of postponing dental visits because they have to fulfill family and job obligations concurrently (e.g., compared to older adults, 65 years and above). The burden increased during the COVID-19 pandemic due to, e.g., school closings and the requirement to work from home. Furthermore, the link between increased affect regarding COVID-19 and postponed dental visits appears plausible. Previous studies have shown a link between dental fear and avoidance of dental visits [17]. It should be noted that (negative) affect is commonly associated with fear or anxiety-related factors [18]. Since there was a lack of studies quantifying the reasons for postponed dental visits, it was difficult to compare our results with studies published in past years.

Postponing dental visits can have serious consequences for oral health. For example, it could result in caries lesions and periodontitis which, in turn, could increase the likelihood of tooth loos [5] or dental pain [19]. This is important, since the COVID-19 pandemic can markedly affect oral health [20,21,22]. Even in the light of the effect of different recall intervals [23], our current findings are therefore of great importance.

This is the first study showing the frequency and correlates of postponed dental visits in Germany during the COVID-19 pandemic. Another strength is that nationally representative data were used. Additionally, the type of postponed dental visits was recorded. One limitation is its cross-sectional design with the acknowledged limitations. Future research is needed to examine postponed dental visits among individuals aged 75 years and older. Moreover, future research is required to explicitly clarify whether the postponed dental visits were postponed by the patient or by the clinician. Furthermore, upcoming studies should include factors such as dental anxiety.

## 5. Conclusions

In conclusion, data showed that more than one out of five individuals postponed a dental visit—particularly check-ups and regular dental examination—due to the COVID-19 pandemic between March and July 2020. Some determinants of these postponed visits have been identified, namely age and affect regarding COVID-19. The findings may help identify and address individuals at risk for deterioration of oral health due to postponed dental visits.

## Figures and Tables

**Table 1:** Sample characteristics for the analytical sample (*n* = 974 individuals) at wave 17.

Independent Variables	Postponed Dental Visits				
	Yes, Postponed Dental Visits	No, Attended as Planned	No Examining Pending	No, other Reasons	*p*-Value
	Mean (SD)/*n* (%)	Mean (SD)/*n* (%)	Mean (SD)/*n* (%)	Mean (SD)/*n* (%)	
Sex					<0.01
Men	91 (19.1%)	127 (26.7%)	240 (50.4%)	18 (3.8%)	
Women	123 (24.7%)	158 (31.7%)	197 (39.6%)	20 (4.0%)	
Age category					<0.01
18 to 29 years	36 (19.1%)	63 (33.3)	83 (43.9%)	7 (3.7)	
30 to 49 years	102 (29.1%)	92 (26.3%)	140 (40.0%)	16 (4.6%)	
50 to 64 years	57 (21.1%)	78 (28.9%)	124 (45.9%)	11 (4.1%)	
65 years and over	19 (11.5%)	52 (31.5%)	90 (54.6%)	4 (2.4%)	
Children under 18 years:					0.07
No	145 (20.1%)	219 (30.3%)	332 (46.0%)	26 (3.6%)	
Yes	69 (27.4%)	66 (26.2%)	105 (41.7%)	12 (4.7%)	
Education					0.53
up to 9 years/10 years and more (without general qualification for university entrance)	88 (19.9%)	132 (29.8%)	206 (46.5%)	17 (3.8%)	
10 years and more (with general qualification for university entrance)	126 (23.7%)	153 (28.8%)	231 (43.5%)	21 (4.0)	
Town size					0.34
Municipality/small town (1–20.000)	80 (19.9%)	128 (31.8%)	174 (43.3%)	20 (5.0%)	
Medium sized town (20.001–100.000)	53 (22.1%)	68 (28.3%)	115 (47.9%)	4 (1.7%)	
Small city (100.001–500.000)	30 (21.1%)	40 (28.2%)	65 (45.8%)	7 (4.9%)	
Big city (> 500.000)	51 (26.8%)	49 (25.8%)	83 (43.7%)	7 (3.7%)	
Region					0.10
West Germany	181 (22.2%)	229 (28.0%)	371 (45.4%)	36 (4.4%)	
East Germany	33 (21.0%)	56 (35.7%)	66 (42.0%)	2 (1.3%)	
Cases/100,000 population					0.40
Below median	109 (23.3%)	142 (30.3%)	197 (42.1%)	20 (4.3%)	
Above median	105 (20.8%)	143 (28.3%)	240 (47.4%)	18 (3.6%)	
Relationship/Marriage					0.35
No	66 (19.6%)	103 (30.5%)	158 (46.9%)	10 (3.0%)	
Yes	148 (23.2%)	182 (28.6%)	279 (43.8%)	28 (4.4%)	
Living situation					0.85
Living alone	54 (21.3%)	73 (28.9%)	118 (46.6%)	8 (3.2%)	
At least 2 individuals in the same household	160 (22.2%)	212 (29.4%)	319 (44.2%)	30 (4.2%)	
Migration background:					0.82
No	183 (22.2%)	236 (28.7%)	372 (45.2%)	32 (3.9%)	
Yes	31 (20.5%)	49 (32.4%)	65 (43.1%)	6 (4.0%)	
Self-employment					0.50
No	196 (22.2%)	252 (28.5%)	400 (45.3%)	35 (4.0%)	
Yes	18 (19.8%)	33 (36.3%)	37 (40.6%)	3 (3.3%)	
Chronic disease					0.20
No	127 (20.9%)	187 (30.7%)	276 (45.3%)	19 (3.1%)	
Yes	87 (23.8%)	98 (26.9%)	161 (44.1%)	19 (5.2%)	
Affect regarding COVID-19 (higher values correspond to higher affect regarding COVID-19)	4.4 (1.0)	4.1 (0.9)	4.1 (1.1)	4.2 (1.1)	<0.001
Presumed severity of COVID-19 infection (from 1 to 7; higher values correspond to higher severity)	4.4 (1.6)	4.1 (1.5)	4.1 (1.6)	4.3 (1.7)	0.09

**Table 2:** Determinants of postponed dental visits (0 = no, not postponed; 1 = yes, postponed) due to the COVID-19 pandemic since March 2020. Findings of multiple logistic regressions.

Independent Variables	Postponed Dental Visits
Gender: Female (Ref.: Male)	1.30
	(0.95–1.79)
Age category: 30 to 49 years (Ref.: 18 to 29 years)	1.42
	(0.87–2.32)
50 to 64 years	0.96
	(0.56–1.63)
65 years and over	0.43 *
	(0.22–0.85)
Children (under 18 years): Yes (Ref.: Absence of children under 18 years)	1.20
	(0.81–1.77)
Education: General qualification for university entrance (Ref.: absence of qualification for university entrance)	1.18
	(0.84–1.65)
Town size: Medium sized town (20,001–100,000) (Ref.: municipality/small town (1–20,000))	1.16
	(0.77–1.74)
Small city (100,001–500,000)	1.09
	(0.67–1.80)
Big city (> 500,000)	1.53 +
	(0.99–2.34)
Region: East Germany (Ref.: West Germany)	0.84
	(0.52–1.35)
Cases/100,000 population: Above median (Ref.: below median)	0.82
	(0.58–1.17)
Relationship/Marriage: Yes (Ref.: no partnership/marriage)	1.17
	(0.76–1.80)
Living situation: At least 2 individuals in the same household (Ref.: living alone)	0.92
	(0.57–1.47)
Migration background: Yes (Ref.: no migration background)	0.85
	(0.54–1.36)
Self-employment: Yes (Ref.: not self-employed)	0.79
	(0.45–1.39)
Chronic disease: Yes (Ref.: no chronic diseases)	1.14
	(0.81–1.61)
Affect: COVID-19 infection (higher values correspond to higher affect)	1.36 **
	(1.13–1.64)
Severity: COVID-19 infection (higher values correspond to higher severity)	1.07
	(0.94–1.22)
Constant	0.04 ***
	(0.01–0.14)
Observations	974
R^2^	0.06

Odds ratios are reported; 95% confidence intervals in parentheses; *** *p* < 0.001, ** *p* < 0.01, * *p* < 0.05, + *p* < 0.10.

## Data Availability

Data are not publicly available but interested parties may contact the authors for more information. The data are not publicly available due to ethical restrictions.

## Supplementary Materials

The following are available online at https://www.mdpi.com/2227-9032/9/1/50/s1, Table S1: Determinants of postponed dental visits due to the COVID-19 pandemic since March 2020. Findings of multiple multinomial logistic regressions (base outcome: Postponed dental visits).
