# Supplementary material for: Postponed Dental Visits during the COVID-19 Pandemic and their Correlates. Evidence from the Nationally Representative COVID-19 Snapshot Monitoring in Germany (COSMO)
Source: Healthcare (Basel). 2021 Jan 5;9(1):50. doi: 10.3390/healthcare9010050 (PMC7824850; doi:10.3390/healthcare9010050)
Supplement: Supplementary file 1 [file healthcare-09-00050-s001.pdf]

Table S1. Determinants of postponed dental visits due to the COVID-19 pandemic since March 2020. Findings of multiple multinomial logistic regressions (base outcome: Postponed dental visits).

**Independent variables**	**No, attended as planned**	**No examining pending**	**No, other reasons**
Gender: Female (Ref.: Male)	0.99<br>(0.68 - 1.44)	0.65*<br>(0.46 - 0.92)	0.81<br>(0.40 - 1.65)
Age category: - 30 to 49 years (Ref.: 18 to 29 years)	0.63<br>(0.36 - 1.11)	0.75<br>(0.44 - 1.27)	0.73<br>(0.25 - 2.15)
- 50 to 64 years	0.92<br>(0.50 - 1.69)	1.16<br>(0.66 - 2.06)	0.86<br>(0.26 - 2.77)
- 65 years and over	2.01+<br>(0.96 - 4.24)	2.69**<br>(1.33 - 5.44)	0.96<br>(0.21 - 4.32)
Children (under 18 years): Yes (Ref.: Absence of children under 18 years)	0.76<br>(0.48 - 1.22)	0.87<br>(0.57 - 1.33)	0.99<br>(0.42 - 2.33)
Education: General qualification for university entrance (Ref.: absence of qualification for university entrance)	0.85<br>(0.57 - 1.25)	0.84<br>(0.59 - 1.21)	0.96<br>(0.46 - 2.02)
Town size: - Medium sized town (20.001 – 100.000) (Ref.: municipality/small town (1-20.000))	0.79<br>(0.49 - 1.26)	0.99<br>(0.64 - 1.53)	0.32*<br>(0.10 - 0.99)
- Small city (100.001 – 500.000)	0.82<br>(0.46 - 1.47)	0.97<br>(0.57 - 1.66)	1.01<br>(0.38 - 2.72)
- Big city (> 500.000)	0.59*<br>(0.35 - 0.98)	0.72<br>(0.45 - 1.14)	0.57<br>(0.22 - 1.48)
Region: East Germany (Ref.: West Germany)	1.52<br>(0.88 - 2.64)	1.13<br>(0.67 - 1.92)	0.26+<br>(0.06 - 1.21)
Cases/100,000 population: Above median (Ref.: below median)	1.20<br>(0.80 - 1.82)	1.29<br>(0.88 - 1.89)	0.72<br>(0.35 - 1.50)
Relationship/Marriage: Yes (Ref.: no partnership/marriage)	0.86<br>(0.52 - 1.41)	0.83<br>(0.52 - 1.32)	1.24<br>(0.46 - 3.30)
Living situation: At least 2 individuals in the same household (Ref.: living alone)	1.10<br>(0.64 - 1.89)	1.08<br>(0.65 - 1.78)	1.19<br>(0.41 - 3.42)
Migration background: Yes (Ref.: no migration background)	1.30<br>(0.76 - 2.21)	1.10<br>(0.66 - 1.81)	1.10<br>(0.40 - 3.01)
Self-employment: Yes (Ref.: not self-employed)	1.61<br>(0.86 - 3.03)	1.08<br>(0.59 - 2.00)	0.99<br>(0.27 - 3.66)
Chronic disease: Yes (Ref.: no chronic diseases)	0.79<br>(0.53 - 1.19)	0.87<br>(0.60 - 1.27)	1.70<br>(0.80 - 3.63)

Affect: COVID-19 infection (higher values correspond to higher affect)	0.72**	0.74**	0.78
	(0.58 - 0.89)	(0.61 - 0.91)	(0.52 - 1.18)
Severity: COVID-19 infection (higher values correspond to higher severity)	0.95	0.92	0.96
	(0.82 - 1.11)	(0.80 - 1.06)	(0.71 - 1.28)
Constant	7.45**	14.94***	3.69
	(1.80 - 30.81)	(3.93 - 56.77)	(0.22 - 62.59)
Observations	974	974	974
R^2	0.04	0.04	0.04

Odds ratios are reported; 95% confidence intervals in parentheses; *** p<0.001, ** p<0.01, * p<0.05, + p<0.10.
